# Tumors with high-density tumor infiltrating lymphocytes constitute a favorable entity in breast cancer: a pooled analysis of four prospective adjuvant trials

**DOI:** 10.18632/oncotarget.6231

**Published:** 2015-10-25

**Authors:** Vassiliki Kotoula, Kyriakos Chatzopoulos, Sotiris Lakis, Zoi Alexopoulou, Eleni Timotheadou, Flora Zagouri, George Pentheroudakis, Helen Gogas, Eleni Galani, Ioannis Efstratiou, Thomas Zaramboukas, Angelos Koutras, Gerasimos Aravantinos, Epaminontas Samantas, Amanda Psyrri, Helen Kourea, Mattheos Bobos, Pavlos Papakostas, Paris Kosmidis, Dimitrios Pectasides, George Fountzilas

**Affiliations:** ^1^ Department of Pathology, Aristotle University of Thessaloniki School of Medicine, Thessaloniki, Greece; ^2^ Laboratory of Molecular Oncology, Hellenic Foundation for Cancer Research, Thessaloniki, Greece; ^3^ Health Data Specialists Ltd, Department of Biostatistics, Athens, Greece; ^4^ Department of Medical Oncology, “Papageorgiou” Hospital, Thessaloniki, Greece; ^5^ Department of Clinical Therapeutics, “Alexandra” Hospital, Athens, Greece; ^6^ Department of Medical Oncology, Ioannina University Hospital, Ioannina, Greece; ^7^ First Department of Medicine, “Laiko” General Hospital, Athens, Greece; ^8^ Second Department of Medical Oncology, “Metropolitan” Hospital, Piraeus, Greece; ^9^ Department of Pathology, “Papageorgiou” Hospital, Thessaloniki, Greece; ^10^ Division of Oncology, Department of Medicine, University Hospital, Patras, Greece; ^11^ Second Department of Medical Oncology, “Agii Anargiri” Cancer Hospital, Athens, Greece; ^12^ Third Department of Medical Oncology, “Agii Anargiri” Cancer Hospital, Athens, Greece; ^13^ Division of Oncology, Second Department of Internal Medicine, Attikon University Hospital, Athens, Greece; ^14^ Department of Pathology, University Hospital of Patras, Rion, Greece; ^15^ Oncology Unit, “Hippokration” Hospital, Athens, Greece; ^16^ Second Department of Medical Oncology, Hygeia Hospital, Athens, Greece; ^17^ Oncology Section, Second Department of Internal Medicine, “Hippokration” Hospital, Athens, Greece

**Keywords:** breast cancer, tumor infiltrating lymphocytes, clinical breast cancer subtypes, trastuzumab, prognostic

## Abstract

**Background:**

Tumor infiltrating lymphocytes (TILs) are considered in the prognosis of breast cancer (BC) patients. Here, we investigated the prognostic/predictive effect of TILs in patients treated in the frame of four prospective trials with adjuvant anthracycline-based chemotherapy in the pre- and post-trastuzumab era.

**Methods:**

TILs density was histologically assessed as percentage of stromal area on whole routine sections of 2613 BC (1563 Luminal A/B; 477 Luminal HER2; 246 HER2-enriched; 327 triple negative [TNBC]) and were evaluated as high/low at three cut-offs (c/o; 50% [lymphocytic predominance, LP], 35% and 25%), in separate training and validation sets.

**Results:**

High TILs were present in 3.5%, 6.5% and 11.5% of all tumors, using the 50%, 35% and 25% c/o, respectively. TILs status did not interact with BC subtypes or trastuzumab treatment. LPBC patient outcome was not affected by nodal status, while high TILs were favorable in TNBC with unfavorable nodal status. When adjusted for standard clinicopathological parameters and treatment, high TILs independently predicted for favorable outcome, e.g., disease-free survival with the 35% c/o in the entire cohort (HR = 0.44, 95% CI 0.28-0.69, *p* < 0.001) and in specific subtypes.

**Conclusions:**

High TILs tumors, especially LPBC seem worthy validating as a separate entity of favorable prognosis in breast cancer.

## INTRODUCTION

Tumor infiltrating lymphocytes (TILs) within the tumor stroma or within tumor nests reflect the host immune response against the tumor, described as cancer “immunoediting” [1]. Measurable tumors are in the “escape” phase during which immune cells are not able to eliminate tumor growth but their presence at least denotes stand-by immunocompetency [1-3] that can be reactivated by treatment. Indeed, in a *per se* non-immunogenic environment, such as the breast, the presence of stromal and/or intratumoral TILs seems to fully support that breast cancer (BC) is immunogenic, especially concerning estrogen receptor (ER) negative disease [3, 4].

Recent studies have shown that TILs presence and density are favorable prognosticators in breast cancer either with [4-11] or without [12] subtype specificity. Similar TILs effects were noticed in the neoadjuvant setting [4, 6, 11, 12] and in the adjuvant setting as well [5, 7, 8, 11], while the presence of TILs in residual disease after initial chemotherapy may also indicate favorable outcome [13]. The presence and density of TILs [7] and the expression signatures of immune function genes [14] may predict benefit from trastuzumab in HER2-positive patients, while TILs presence may also be predictive for benefit from cytotoxic drugs like docetaxel [8] and carboplatin [6].

Methods for TILs assessment in breast cancer greatly vary in different studies, ranging from simple mononuclear cell infiltrate counting on routine hematoxylin & eosin (H&E) stained slides [7, 8, 15]; immune cell typing with immunohistochemistry [11, 12, 15]; and, immune-related gene expression [4, 9, 14] to digital immune cell weighing [16]. In an effort to bring TILs closer to clinical application, recommendations for the morphological assessment of TILs on H&E sections have also been recently published [17].

In the present pooled analysis, we investigated the effect of TILs on the outcome of more than 2500 patients with operable breast cancer who were treated in the setting of prospective trials conducted by the Hellenic Cooperative Oncology Group (HeCOG). The study population allowed for the evaluation of TILs as prognostic parameter upon anthracycline - taxane regimens and as predictive for trastuzumab benefit in HER2 positive patients. TILs were morphologically assessed as a continuous variable but were finally analyzed at three different cut-offs according to breast cancer clinical subtypes and to nodal status.

## RESULTS

**Figure 1:**
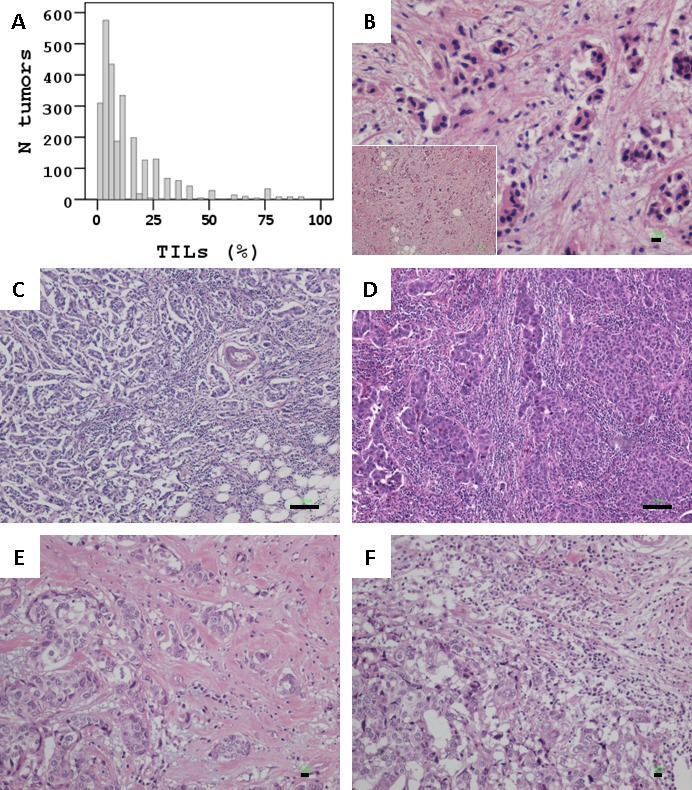
Stromal TILs in breast carcinomas **A.** Distribution of TILs rates in the study population. **B.** < 5% (Bar: 10um); **C.** 50% (Bar: 100um); **D.** 75% (Bar: 100um) TILs homogeneously dispersed in these tumors. E and F: Same case, overall 35% TILs, heterogeneously populating the stroma of this tumor. E at 10%, F at 40%. Bars at 10um.

The distribution of TILs rates is shown in Figure 1A. The mean stromal TILs density in all 2618 tumors was 12.7 (±SD 15.5) and the median was 7. In 1290 tumors (49.3% of total), stromal TILs rate was 0 - 5%, while additional 587 tumors (22.4%) had 10 - 20% stromal TILs.

Among all tumors, 91 (3.5%) were considered as LPBC since they had ≥50% stromal TILs; 170 (6.5%) had ≥35%, and 301 (11.5%) had ≥25% TILs. In LPBC and in tumors with TILs close to 0 (Figure 1B-1D), these infiltrates were homogeneously dense or absent in the same tumor, respectively. In comparison, TILs distribution was heterogeneous in cases with 5 - 35%, i.e., TILs density differed by >10% in different areas in the same section or among sections from the same tumor (Figure 1E & 1F).

### TILs and clinicopathological characteristics

**Figure 2:**
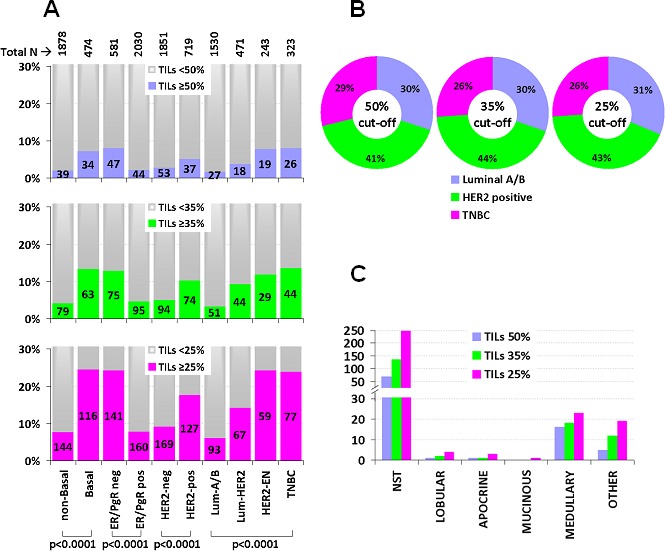
TILs association with IHC breast cancer phenotypes and with specific histological types **A.** Numbers in bars indicate the actual population per category. High TILs bars (blue, green, violet) are shown as percentage per phenotype with all examined cut-offs, as indicated. A prevalence of high TILs in the non-ER/PgR context is evident, where the incidence of tumors with ≥50% (lymphocyte predominant, LPBC) was minimal. **B.** By contrast, the incidence of major subtypes within the group of high TILs tumors was not substantially different (chi square *p* = 0.87). **C.** Numbers in the Y-axis represent the actual population per category. The majority of high TILs tumors were of the non-specific type (NST). However, the rate of high TILs tumors among the 24 medullary carcinomas was the highest among all subtypes. Lobular, apocrine and mucinous carcinomas seldom exhibited high-TILs.

Higher stromal TILs rates, either as continuous or binary variables as shown in Figure 2A, were significantly more frequent in the absence of ER/PgR, in HER2 positive, especially in HER2-enriched, in basal-like and in TNBC tumors (all p's < 0.0001). For example, with the 35% cut-off, TNBC (13.6%), HER2-enriched (11.9%) and Luminal HER2 (9.3%) had significantly more often high TILs as compared to Luminal A/B (3.3%) tumors. Importantly though, Luminal A/B, HER2-positive and TNBC were represented at similar rates within the group of tumors with high TILs (Figure 2B).

Tumors with high TILs also had statistically significantly higher Ki67 (Mann-Whitney *p* < 0.001) and were more often of grade III (Pearson's chi-square *p* < 0.001). However, Ki67 labeling values largely overlapped between high and low TILs categories, rendering the biological significance of this finding questionable. TILs density was not associated with nodal status and tumor size in our series; it also did not differ in comparison to age and menopausal status (Table S5).

With respect to specific histological breast cancer types that were adequately represented in the studied cohort for statistical comparisons (>15 cases per subtype), high TILs were present in the majority of the 24 medullary carcinomas (67%, 75%, and 96% with the 50%, 35% and 25% TILs cut-offs, respectively), while these were absent or very rare among the 221 lobular, 30 apocrine, and 17 mucinous carcinomas (Figure 2C).

### Favorable prognostic high TILs in breast cancer

**Figure 3:**
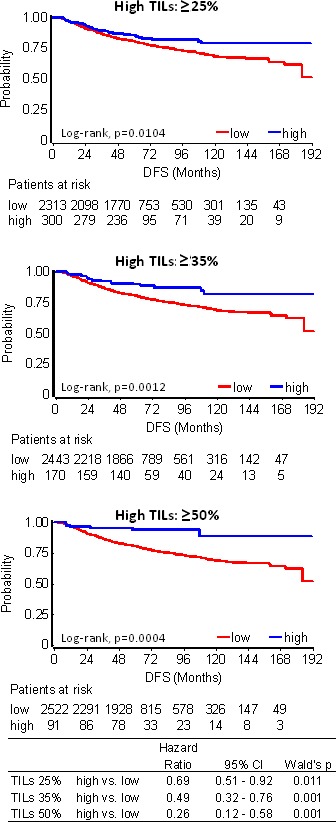
TILs association with patient disease-free survival (DFS) in 2613 breast cancer patients High TILs at all cut-offs examined conferred longer DFS.

Among all patients, even without distinguishing for subtype and trastuzumab treatment, high TILs at all examined cut-offs were associated with longer DFS as compared to low TILs (Figure 3). Among the 91 patients with LPBC (TILs ≥50%), only 6 (6.6%) relapsed during a period of more than 10yrs follow-up and out of them only 2 (2.2% of all LPBC) during the first 3 years (Table 1). None or rare events were noticed for these patients for overall survival (OS) as well (Table S6). Risk for relapse and death was remarkably constant in the training and validation sets; however, due to the small number of patients and events (0-2 patients with high TILs tumors in subtype categories), results in the validation set appeared as statistically non-significant. Similar, albeit less pronounced results were obtained for favorable high TILs at the 35% and 25% cut-offs in the entire patient cohort (Table 1).

**Table 1:** Univariable analyses (Log-rank and Cox) showing the effect of high TILs at all 3 cut-offs in the training and validation sets with respect to patient disease-free survival

Patient group		No of patients	No of events	Hazard Ratio	95% CI	% event free at 3-years	% event free at 5-years	% event free at 7-years	Log-rank p	Wald's p
		**TIL cut-off 50%, high vs. low**								
**Whole dataset**	**All patients**	91 vs. 2527	6 vs. 595	0.26	0.12 - 0.58	95.5% vs. 86.9%	94.0% vs. 80.8%	94.0% vs. 75.6%	<0.001	0.001
	**Luminal A/B**	27 vs. 1536	2 vs. 321	0.35	0.09 - 1.39	96.3% vs. 89.4%	90.6% vs. 83.5%	90.6% vs. 78.1%	0.118	0.136
	**HER2-positive**	37 vs. 686	3 vs. 186	0.27	0.09 - 0.86	94.4% vs. 85.2%	94.4% vs. 78.2%	94.4% vs. 71.4%	0.017	0.026
	**TNBC**	27 vs. 300	1 vs. 85	0.12	0.02 - 0.85	96.3% vs. 77.9%	96.3% vs. 74.0%	96.3% vs. 72.8%	0.011	0.034
**Training set**	**All patients**	47 vs. 1250	2 vs. 312	0.16	0.04 - 0.63	97.9% vs. 86.0%	97.9% vs. 80.4%	97.9% vs. 73.4%	0.003	0.009
	**Luminal A/B**	16 vs. 769	1 vs. 167	0.33	0.05 - 2.36	90.0% vs. 90.4%	90.0% vs. 84.4%	90.0% vs. 77.7%	0.397	0.270
	**HER2-positive**	14 vs. 331	1 vs. 100	0.18	0.03 - 1.32	100.0% vs. 82.7%	100.0% vs. 76.7%	100.0% vs. 67.8%	0.058	0.091
	**TNBC**	17 vs. 146	0 vs. 43	0.09	0.01-1.44	100.0% vs. 75.3%	100.0% vs. 73.3%	100.0% vs. 70.9%	0.016	0.090
**Validation set**	**All patients**	44 vs. 1277	4 vs. 283	0.39	0.15 - 1.05	93.0% vs. 87.8%	89.9% vs. 81.3%	89.9% vs. 77.7%	0.052	0.061
	**Luminal A/B**	11 vs. 767	1 vs. 154	0.37	0.05 - 2.67	100.0% vs. 88.5%	90.0% vs. 82.7%	90.0% vs. 78.6%	0.167	0.327
	**HER2-positive**	23 vs. 355	2 vs. 86	0.38	0.09 - 1.54	90.7% vs. 87.6%	90.7% vs. 79.5%	90.7% vs. 74.9%	0.158	0.174
	**TNBC**	10 vs. 154	1 vs. 42	0.34	0.05 - 2.47	90.0% vs. 80.4%	90.0% vs. 74.7%	90.0% vs. 74.7%	0.262	0.285
		**TIL cut-off 35%, high vs. low**								
**Whole dataset**	**All patients**	170 vs. 2448	21 vs. 580	0.49	0.32 - 0.76	92.3% vs. 86.8%	89.7% vs. 80.7%	87.1% vs. 75.4%	0.001	0.001
	**Luminal A/B**	52 vs. 1511	6 vs. 317	0.55	0.25 - 1.24	94.2% vs. 89.3%	89.8% vs. 83.4%	86.8% vs. 78.0%	0.143	0.149
	**HER2-positive**	73 vs. 650	10 vs. 179	0.46	0.24 - 0.87	93.0% vs. 84.9%	90.2% vs. 77.7%	87.0% vs. 71.0%	0.014	0.016
	**TNBC**	45 vs. 282	5 vs. 81	0.36	0.15 - 0.88	88.9% vs. 77.9%	88.9% vs. 73.8%	88.9% vs. 72.5%	0.020	0.025
**Training set**	**All patients**	88 vs. 1209	9 vs. 305	0.38	0.19 - 0.73	94.3% vs. 85.8%	92.0% vs. 80.2%	90.2% vs. 73.2%	0.003	0.004
	**Luminal A/B**	32 vs. 753	4 vs. 164	0.62	0.23 - 1.68	88.0% vs. 90.4%	84.0% vs. 84.5%	78.7% vs. 77.9%	0.960	0.350
	**HER2-positive**	33 vs. 312	4 vs. 97	0.32	0.12 - 0.87	93.9% vs. 82.3%	90.9% vs. 76.2%	90.9% vs. 66.8%	0.019	0.026
	**TNBC**	23 vs. 140	1 vs. 42	0.13	0.02 - 0.91	95.7% vs. 75.0%	95.7% vs. 72.8%	95.7% vs. 70.3%	0.015	0.040
**Validation set**	**All patients**	82 vs. 1239	12 vs. 275	0.64	0.36 - 1.14	90.1% vs. 87.8%	87.2% vs. 81.2%	84.1% vs. 77.7%	0.129	0.132
	**Luminal A/B**	20 vs. 758	2 vs. 153	0.45	0.11 - 1.82	100.0% vs. 88.3%	94.7% vs. 82.5%	94.7% vs. 78.3%	0.045	0.262
	**HER2-positive**	40 vs. 338	6 vs. 82	0.63	0.27 - 1.44	92.2% vs. 87.3%	89.6% vs. 79.1%	82.7% vs. 74.9%	0.267	0.272
	**TNBC**	22 vs. 142	4 vs. 39	0.64	0.23 - 1.78	81.8% vs. 80.9%	81.8% vs. 74.7%	81.8% vs. 74.7%	0.384	0.388
		**TIL cut-off 25%, high vs. low**								
**Whole dataset**	**All patients**	301 vs. 2317	50 vs. 551	0.68	0.51 - 0.91	89.3% vs. 86.9%	85.7% vs. 80.7%	81.9% vs. 75.4%	0.009	0.009
	**Luminal A/B**	95 vs. 1468	17 vs. 306	0.87	0.53 - 1.42	88.4% vs. 89.6%	83.8% vs. 83.7%	80.1% vs. 78.2%	0.578	0.578
	**HER2-positive**	127 vs. 596	22 vs. 167	0.59	0.38 - 0.92	91.2% vs. 84.5%	87.1% vs. 77.3%	81.5% vs. 70.7%	0.017	0.019
	**TNBC**	78 vs. 249	11 vs. 75	0.45	0.24 - 0.84	87.2% vs. 77.0%	85.9% vs. 72.8%	85.9% vs. 71.4%	0.010	0.012
**Training set**	**All patients**	155 vs. 1142	27 vs. 287	0.68	0.46 - 1.00	89.0% vs. 86.0%	85.0% vs. 80.5%	81.5% vs. 73.4%	0.049	0.051
	**Luminal A/B**	57 vs. 728	13 vs. 155	1.2	0.68 - 2.12	83.7% vs. 90.8%	79.6% vs. 84.8%	73.1% vs. 78.3%	0.463	0.525
	**HER2-positive**	57 vs. 288	10 vs. 91	0.5	0.26 - 0.95	89.5% vs. 82.2%	85.7% vs. 76.0%	82.9% vs. 66.5%	0.032	0.036
	**TNBC**	40 vs. 123	4 vs. 39	0.28	0.10 - 0.78	92.5% vs. 73.2%	89.9% vs. 71.5%	89.9% vs. 68.7%	0.009	0.015
**Validation set**	**All patients**	146 vs. 1175	23 vs. 264	0.68	0.45 - 1.05	89.6% vs. 87.7%	86.6% vs. 81.0%	82.6% vs. 77.5%	0.079	0.081
	**Luminal A/B**	38 vs. 740	4 vs. 151	0.46	0.17 - 1.25	93.5% vs. 88.4%	88.3% vs. 82.6%	88.3% vs. 78.3%	0.126	0.127
	**HER2-positive**	70 vs. 308	12 vs. 76	0.7	0.38 - 1.28	92.7% vs. 86.7%	88.2% vs. 78.4%	80.1% vs. 74.7%	0.238	0.242
	**TNBC**	38 vs. 126	7 vs. 36	0.66	0.29 - 1.48	81.6% vs. 80.8%	81.6% vs. 74.0%	81.6% vs. 74.0%	0.310	0.313

**Figure 4:**
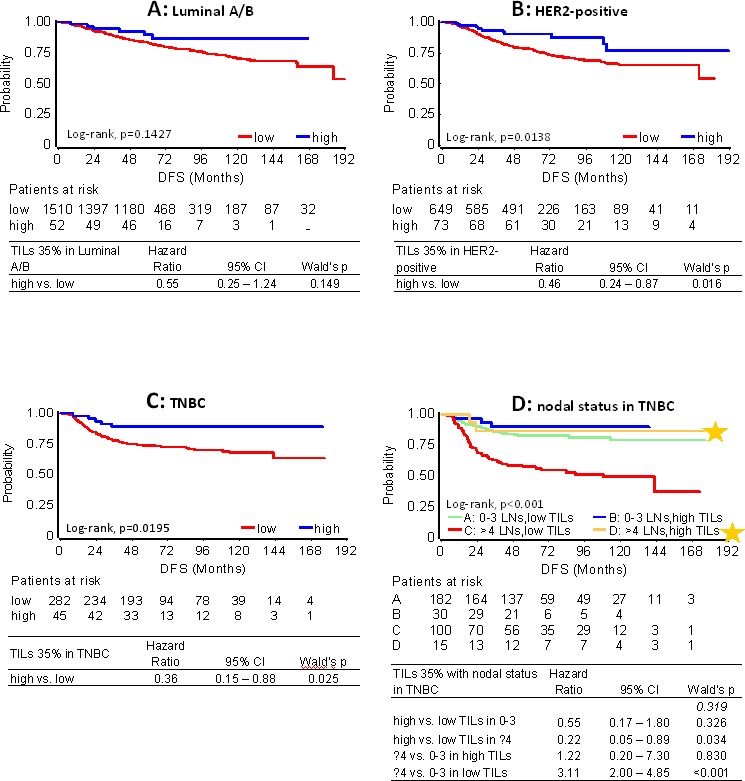
Effect of high TILs on patient DFS with respect to clinical breast cancer subtypes Results are shown for high TILs at the 35% cut-off. In all subtype categories **A.** - **C.** high TILs were associated with longer DFS. In **A.** although high TILs are not considered prognostic in Luminal A/B disease, patients with such tumors who did not relapse within the first 5 years remained relapse free for more than 15 years. In **B.** HER2-positive patients were treated with and without trastuzumab. **D.** Patients with unfavourable nodal status and high TILs (yellow star) fared equally well as patients with favourable nodal status, where TILs levels were not associated with outcome.

As can be retrieved from Table 1, the rate of relapses for high TILs at any cut-off was similarly low among subtypes. However, when comparing the impact of TILs density with respect to each subtype, no statistically significant effect was observed for high TILs on the outcome of patients with Luminal A/B tumors. Still, such patients with high TILs, for example at the 35% cut-off, who did not relapse within the first 5 yrs (46 out of 52) remained relapse-free during the entire follow-up period (Figure 4A, Table 1). High TILs at all cut-offs conferred decreased risk for relapse and death in patients with HER2 positive tumors irrespectively of trastuzumab treatment (Figure 4B). This effect was particularly strong in LPBC where only 3/37 patients relapsed and none died within the first 3 years from diagnosis, as compared to 101/686 relapses and 30 deaths that were observed for non-LPBC HER2-positive patients during the same period (Table 1). Similarly, favorable high TILs effects at all cut-offs, again more pronounced for LPBC were observed for TNBC patients (Figure 4C). Only 1 out of 27 TNBC patients with LPBC relapsed and none died during the entire follow-up period, as compared to 85 and 71 out of the 300 non-LPBC TNBC patients who relapsed and died, respectively (Table 1).

In the entire cohort and with respect to clinical subtypes, high nodal burden (≥4 infiltrated lymph nodes) strongly predicted for shorter DFS and OS as compared to low nodal burden (0-3 nodes) (Table S7). High nodal burden was universally unfavorable for patients with low TILs tumors at all cut-offs (Table S8a for DFS; Table S8b for OS). In all settings, nodal status did not affect the outcome of LPBC patients, for whom 0 to 1 relapses were observed per subtype group. For patients with >35% and >25% TILs, high nodal burden remained unfavorable in the Luminal A/B and HER2-positive groups. Of note, the rare events in the favorable high TILs groups always concerned the same Luminal A/B and HER2-positive patients, which probably biased the statistical significance of the interaction for the 25% cut-off. In TNBC patients, high TILs conferred significantly better outcome for unfavorable nodal status (Figure 4D), other than in the Luminal A/B and HER2-positive groups. Again, however, the numbers of events were very small and no statistical significance was reached for this interaction.

Histologic subtypes were not separately evaluated for TILs effect on outcome, due to small numbers in each category.

### High TILs did not significantly predict for trastuzumab benefit

**Figure 5:**
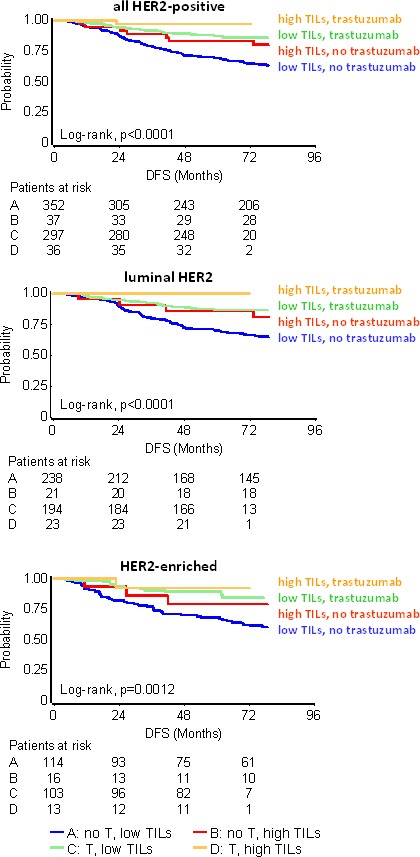
Effect of TILs with respect to trastuzumab (T) in HER2-positive patients Results are shown for high TILs at the 35% cut-off. Whether statistically significant or not, high TILs were favourable in every context examined (all HER2-positive and HER2-positive subtypes). Trastuzumab significantly benefitted patients with low TILs but this effect, although present, was insignificant for patients with high TILs.

The effect of TILs in HER2 positive disease was also examined as an interaction with trastuzumab, by comparing the outcome of patients treated in the pre- and post-trastuzumab era. Among all HER2-positive patients, a significant trastuzumab benefit was noticed for those with low TILs. Thus, trastuzumab significantly benefited patients with < 35% TILs (HR: 0.37, 95%CI: 0.25-0.53), with < 25% TILs (HR: 0.39, 95%CI: 0.27-0.57), and, with < 50% TILs (HR: 0.36, 95%CI 0.25-0.52). In comparison, trastuzumab did not offer statistically significant outcome advantage in patients with high TILs tumors; with or without the drug, patients with high TILs fared better than those with low TILs (Table 2 and Figure 5 for the 35% cut-off; Table S9 [DFS] and Table S10 [OS] for LPBC and for the 25% cut-off). No interaction between trastuzumab and TILs was found among all HER2-positive patients (Figure 5A). Similar results were obtained for all TILs cut-offs, in the entire group of HER2-positive patients and separately in the test and validation sets for DFS (Table S9) and OS (Table S10). However, at all comparisons, HER2-positive patients treated with trastuzumab for tumors with high TILs density fared best. For example, none of 18 trastuzumab-treated patients with LPBC and only 2/61 patients with >25% TILs relapsed over a period of 5 years, as compared to 2/19 and 18/66 non-treated patients with respective TILs status (Table S9). In addition, no interaction was observed between TILs density and HER2-positive subtypes (Figure 5B and 5C), despite that trastuzumab treated patients with high TILs fared best with few relapses during the entire follow-up period. Relapse rate was proportionally lower for patients with high TILs Luminal-HER2 as compared to those with HER2-Enriched, but for both subtypes high TILs were favorable as compared to low TILs. Again, statistical results were probably biased by the small number of patients and events for each HER2-positive subtype with high TILs (Table 2).

**Table 2:** Interaction tests for TILs, T treatment and HER2 positive subtypes on patient DFS

	No of patients	No of events	Hazard Ratio	95% CI	Wald's p
***TILs cut-off 35%, training set***					
**TILs interaction with T treatment**					0.584
T Yes vs. No at low TILs	148 vs. 164	24 vs. 63	0.45	0.28 - 0.72	0.001
T Yes vs. No at high TILs	15 vs. 18	0 vs. 3	0.19	0.01 - 3.82	0.277
TILs high vs. low at T No	18 vs. 164	3 vs. 63	0.43	0.15 - 1.29	0.124
TILs high vs. low at T Yes	15 vs. 148	0 vs. 24	0.19	0.01 - 3.16	0.261
**TILs interaction with HER2-positive subtypes**					0.935
Luminal HER2 vs. HER2-Enriched at low TILs	202 vs. 110	52 vs. 35	0.77	0.50 - 1.19	0.24
Luminal HER2 vs. HER2-Enriched at high TILs	24 vs. 9	2 vs. 1	0.86	0.08 - 9.44	0.909
TILs high vs. low at HER2-Enriched	9 vs. 110	1 vs. 35	0.27	0.04 - 1.99	0.19
TILs high vs. low at Luminal HER2	24 vs. 202	2 vs. 52	0.3	0.07 - 1.24	0.1
***TILs cut-off 35%, validation set***					
**TILs interaction with T treatment**					0.841
T Yes vs. No at low TILs	149 vs. 189	14 vs. 63	0.28	0.16 - 0.51	<0.001
T Yes vs. No at high TILs	21 vs. 19	1 vs. 4	0.22	0.03 - 2.01	0.159
TILs high vs. low at T No	19 vs. 189	4 vs. 63	0.64	0.23 - 1.75	0.396
TILs high vs. low at T Yes	21 vs. 149	1 vs. 14	0.51	0.07 - 3.84	0.52
**TILs interaction with HER2-positive subtypes**					0.434
Luminal HER2 vs. HER2-Enriched at low TILs	231 vs. 107	55 vs. 22	1.1	0.67 - 1.81	0.72
Luminal HER2 vs. HER2-Enriched at high TILs	20 vs. 20	2 vs. 3	0.53	0.09 - 3.15	0.494
TILs high vs. low at HER2-Enriched	20 vs. 107	3 vs. 22	0.8	0.24 - 2.69	0.731
TILs high vs. low at Luminal HER2	20 vs. 231	2 vs. 55	0.38	0.09 - 1.57	0.186
***TILs cut-off 35%, whole dataset***					
**TILs interaction with T treatment**					0.427
T Yes vs. No at low TILs	297 vs. 353	38 vs. 126	0.37	0.25 - 0.53	<0.001
T Yes vs. No at high TILs	36 vs. 37	1 vs. 7	0.16	0.02 - 1.26	0.083
TILs high vs. low at T No	37 vs. 353	7 vs. 126	0.49	0.23 - 1.06	0.067
TILs high vs. low at T Yes	36 vs. 297	1 vs. 38	0.21	0.03 - 1.52	0.119
**TILs interaction with HER2-positive subtypes**					0.576
Luminal HER2 vs. HER2-Enriched at low TILs	433 vs. 217	107 vs. 57	0.9	0.65 - 1.24	0.533
Luminal HER2 vs. HER2-Enriched at high TILs	44 vs. 29	4 vs. 4	0.6	0.15 - 2.39	0.479
TILs high vs. low at HER2-Enriched	29 vs. 217	4 vs. 57	0.51	0.19 - 1.41	0.189
TILs high vs. low at Luminal HER2	44 vs. 433	4 vs. 107	0.34	0.13 - 0.92	0.03

T: trastuzumab

Finally, no interaction was observed between TILs density, disease stage I vs. II, and nodal status with respect to trastuzumab treatment.

### High TILs as an independent favorable prognostic marker in breast cancer

**Figure 6:**
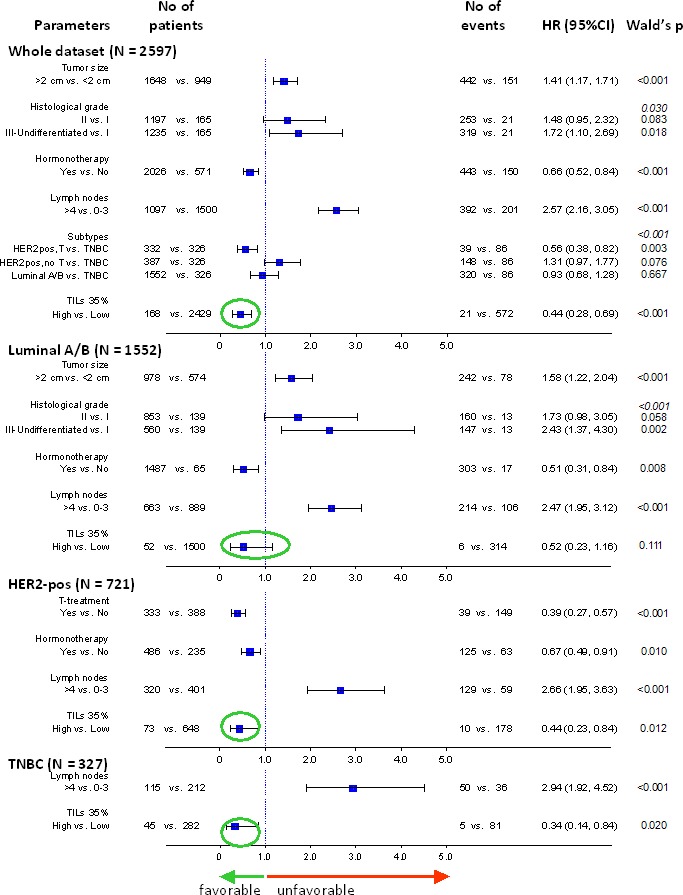
Forest plot showing the strongly significant independent favourable effect of high TILs on patient DFS Multivariable models for the entire cohort and for breast cancer subtypes are shown. High TILs, here shown according to the 35% cut-off, had a constantly favourable effect on patient outcome in all settings (green circles).

Multivariable models including standard clinicopathological parameters affecting patient outcome, as described in the Methods section and in Table S7, revealed the independent favorable prognostic effect of high TILs in breast cancer patients treated with adjuvant anthracyclines and taxanes, as shown for DFS in Figure 6 and for OS in Table S11. In all models examined for each subtype separately, high TILs retained their favorable independent prognostic effect. When examined in the entire patient population, high TILs were the strongest favorable prognosticator in the examined clinical context.

## DISCUSSION

This study confirmed the presence of high TILs as a robust favorable prognosticator in high-risk operable breast cancer, an effect that was more pronounced in HER2 positive and TNBC patients, in line with previous reports in the adjuvant [5, 7-9, 11, 24] and neoadjuvant setting [6, 9, 11, 13]. Herein, instead of using continuous TILs counts and reporting on added risk discounts, TILs were evaluated in a binary mode as high/low according to predefined cut-offs, all of which were validated in a second study set and in the entire population. These data and the fact that the patients studied represented a pooled cohort from different prospective trials that were conducted over a period spanning more than 10 years of clinical practice, support the robustness of morphologically assessed high TILs as a favorable prognostic marker in operable high-risk breast cancer, definitely in tumors with unfavorable subtypes.

In the better prognosis Luminal A/B tumors, high TILs may also offer DFS advantage; however, even with the present long follow-up, statistics were underpowered for the evaluation of high TILs in this group of patients. High TILs tumors, even at the lowest cut-off we applied, are an infrequent occasion in breast cancer, with lowest rates in Luminal A/B; the latter constitutes an additional reason for low statistical power and for inconclusive results in this group of tumors in most studies, as also recently noticed [24]. However, if we consider high TILs tumors as a separate entity the following apply: (a) major subtypes, i.e., Luminal A/B, HER2 positive irrespective of ER/PgR, and, TNBC are evenly distributed within this entity; (b) the rate of events per subtype within the entity is very similar; (c) the number of events is extremely limited for cases with LPBC. By setting the cut-off for calling LPBC at 50% TILs density, corresponding patients of any subtype had an excellent course of disease, as previously described for HER2 positive and TNBC [7, 9]. In our series, about 10% of patients with non-Luminal A/B tumors and less than 2% of patients with Luminal A/B had LPBC. Due to the high worldwide incidence of breast cancer, however, the corresponding absolute number of patients is not negligible. Our data support addressing LPBC as a distinct breast cancer subtype to be considered for alternative therapeutic interventions.

In the presented series, the cohort specific cut-off for distinguishing high/low TILs was 35%, while the favorable TILs effect statistically disappeared below 25% average density. This gradual declining of prognostic TILs significance supports the notion to evaluate TILs as a continuous parameter [17], which, however, may be impractical for the application of this marker in diagnostics. Although the need to consistently report TILs still remains [25], based on our findings, a three-scale grading system for reporting TILs status seems applicable, i.e., LPBC (high and homogeneous), non-LPBC with high TILs called at 25% as the lower cut-off, and non-LPBC with TILs below that level.

Of note, a parameter that may also need to be assessed along with TILs density is the heterogeneous distribution of TILs in the stroma of breast carcinomas. As shown here, apart from LPBC and from tumors with close to null immune infiltrates, TILs density is largely heterogeneous within the same tumor, which is challenging to report in routine diagnostics. Assessing the degree or patterns of spatial intratumoral TILs heterogeneity might also be an option for a more detailed description of the immune response status of the tumor, as recently suggested for ER/PgR negative breast cancer [16]. In order to approach the heterogeneous TILs distribution, we may consider anti-tumor immune response being triggered by neo-antigens produced by the tumor, which is the result of the underlying genetic alterations [26]. Following this concept, the observed spatial heterogeneity in TILs density may reflect the well established genetic heterogeneity in breast cancer. Although the issue is tremendously important, such data from clinical material are still missing. Immune response to neoantigens is likely a universal process, not confined to specific breast cancer subtypes or to clinically actionable targets, at least not in the way these parameters are currently considered. The fact that the majority of high TILs tumors are found among TNBC and HER2-positive cases may just reflect the extensive genomic alterations described for these tumors as compared to Luminal A/B [27]. Since, as described here, heterogeneous TILs density is observed in non-LPBC, it seems worthy including TILs heterogeneity as a binary parameter (presence vs. absence) for the evaluation of non-LPBC in future studies.

With respect to HER2-positive tumors, we did not observe any statistically significant interaction between TILs and trastuzumab treatment. Trastuzumab significantly benefitted patients with low TILs as determined here; patients with high TILs fared better with or without this drug. This appears in line with the findings by Perez et al for the N9831 trial [28] but in contrast to the previously reported predictive role of TILs for trastuzumab benefit in the FinHER trial [7]. In this trial, HER2-positive LPBC without trastuzumab fared worse than non-LPBC, which was not the case in our series from the pre-trastuzumab era involving a larger number of patients; this may explain the present discrepant statistical result with respect to the predictive role of TILs for trastuzumab benefit. Further, the same prognostic effect of high TILs was observed with all cut-offs used herein. However, although not statistically significant, patients with high TILs who were treated with trastuzumab fared best over a period of 5 years. Overall, these results support the prognostic role of high TILs in HER2-positive disease independently of trastuzumab treatment and underline the necessity for larger patient series and follow-up longer than 5 years for the evaluation of the predictive value of TILs with respect to trastuzumab.

A novel piece of data in the present study concerns the impact of nodal status in association with TILs density on patient outcome. Universal findings for all subtypes at all TILs cut-offs were (a) that patients with LPBC fared best irrespectively of nodal status, which further indicates LPBC as a distinct entity; and (b) that the aggravating prognostic impact of low vs. high nodal burden was effective in patients with low TILs tumors, which was constant in all comparisons. Inefficiency of the host to block tumor expansion may only partially explain this condition, since, for example, patients with high TILs TNBC and unfavorable nodes treated with adjuvant chemotherapy fared as well as patients with favorable nodes. As reported, immune changes in tumor draining lymph nodes may not be solely driven by local tumor invasion [29] but the biological basis of the role of intratumoral TILs in the behavior of locally spread disease has not yet been investigated. Nevertheless, it remains challenging and clinically important to dissect patient prognosis beyond the classic parameter of nodal status. If the present findings are validated in independent studies TILs density may be helpful in this context.

In conclusion, the presented pooled analysis supports the need for the histological assessment of TILs density as a major marker of outcome along with nodal status in breast cancer patients treated in the adjuvant setting. No clear predictive role was revealed for trastuzumab benefit with this marker but it is useful to know that trastuzumab-treated patients with high TILs fare best. Distinguishing LPBC as a distinct entity with excellent prognosis in the adjuvant setting appears clinically important for treatment decisions, while using high TILs cut-offs may identify patients who would benefit from agents recharging their anti-tumor immune response. The present findings merit validation in independent large cohorts by taking into account the low rate of high TILs tumors in patients with operable high-risk breast cancer.

## MATERIALS AND METHODS

### Patients and tumors

Tumor tissue material from 2618 patients who had been diagnosed between 1997 and 2010 with operable breast cancer and had been treated with adjuvant chemotherapy (anthracyclines - taxanes) in the setting of four prospective clinical trials by the Hellenic Cooperative Oncology Group (HeCOG) was examined (HE 10/97 [18]; HE 10/00 [19]; HE 10/05 [20]; HE 10/08 [manuscript in preparation]). The basic trial characteristics are shown in Table S1. In HE10/05 and HE10/08 trastuzumab was administered sequentially for one year after the completion of chemotherapy. Patients had provided written consent for the use of their biologic material for research purposes and the study was approved by the Bioethics Committee of the Aristotle University of Thessaloniki School of Medicine (#77/10June2014) and by the Institutional Review Board of Papageorgiou Hospital of Thessaloniki (#725/10May2013). Paraffin blocks were collected retrospectively for HE10/97 and prospectively for the other 3 trials. The distribution of patients and tumors per clinical study and basic demographic and clinicopathological characteristics are shown in Table S2.

Tumors had been routinely diagnosed in local pathology labs, where they were also evaluated for ER/PgR/HER2 protein expression with immunohistochemistry (IHC) for patient stratification to receive hormone treatment and trastuzumab. HER2 FISH or CISH had also been applied locally in cases with ambiguous HER2 IHC for patients in trials HE 10/05 and HE 10/08. HER2-positive patients were treated with trastuzumab in these two trials (post-trastuzumab era) but not in HE 10/97 and HE 10/00 (pre-trastuzumab era). For the purposes of the present study, tumors were evaluated for combined ER/PgR positivity in the absence of HER2 protein overexpression and/or gene amplification as Luminal A/B tumors; Luminal-HER2 if ER/PgR positive and HER2 over-expressing or amplified; HER2-enriched if ER/PgR negative but HER2 pathology positive; and, as triple negative (TNBC) if ER/PgR/HER2 negative.

All tumors were also retrospectively centrally subtyped in the Laboratory of Molecular Oncology (HeFCR/HeCOG/AUTH, Thessaloniki, GR) with ER/PgR/HER2/Ki67 IHC and HER2 FISH, as previously described [21]. In addition, cytokeratin-5 (CK5) and epidermal growth factor receptor (EGFR) IHC were applied for typing basal-like carcinomas with 1% cut-off for positivity, as suggested [22].

### TILs evaluation

Mononuclear infiltrates corresponding to TILs were assessed on whole routine hematoxylin and eosin (H&E) sections of 2613 breast carcinomas, by one pathologist (K.Ch.) as % of stromal area according to the recently published recommendation [17]. In 5 cases it was impossible to evaluate the stromal component of the tumor. Areas to be evaluated were identified under low power (X100), TILs morphology was validated under higher power, and TILs density was assessed as % of covered stromal area under low power if >50% and under higher power (X200) if very low and up to 50%. Efforts were paid to exclude intra-tumoral TILs but this distinction was not always possible due to tumor architecture patterns. Sections from all available blocks per case were evaluated (2 blocks in 43 and 3 blocks in 16 cases; all others, 1 block per case). The entire sections were scanned and average TILs content was recorded per case. Tertiary lymphoid structures, areas with in situ carcinoma or lobular cancerization that often exhibited regional high TILs, and necrotic areas were not considered. Semi-continuous values (5% increments) were recorded per case. Representative examples of the range of TILs densities encountered in the present study are given in Figure 1.

### Statistical analysis

Categorical variables were presented as frequencies and percentages while various measures (mean, standard deviation, median, range) were used for continuous variables. Hormonotherapy and trastuzumab were administered based on local breast cancer typing; therefore, local tumor classification based on ER/PgR and HER2 status (IHC/FISH or CISH) was used for analysis in the present study. Concordance for calling HER2 positive tumors and TNBC between local and central pathology was 90.6% (Cohen's Kappa = 53.1, 95%CI = 47.7-58.5) and 86.9% (Cohen's Kappa = 64.6, 95%CI = 61.0-68.2), respectively.

Due to the exploratory nature of the study, no correction for multiple testing was applied. In order to assess the reproducibility/validity of the outcome analysis findings, the whole dataset was split into training and validation sets in a controlled fashion. For the latter purpose, nodal status, tumor size, menopausal status, hormone therapy and treatment with trastuzumab were equally assigned into both sets. Patient characteristics did not significantly differ between the two sets (Table S3).

Survival status was updated in June 2014. Disease-free survival (DFS) was set as the primary endpoint of the study and overall survival (OS) as the secondary. DFS was measured from the date of diagnosis until verified disease progression, death or last contact, whichever occurred first, while OS from diagnosis until death from any cause or date of last contact. The annual hazard function for the risk of recurrence (DFS) was estimated by subtypes using penalized B-splines. The number of knots used for the splines was 100. The estimated hazards are presented along with the 95% CIs (Table S4).

There is currently no standard cut-off for TILs classification in BC, while it is suggested that TILs be examined in a continuous mode [17]. Unlike previous reports using 10% increments of recorded TILs values for outcome comparisons (e.g. [7]), here we used three fixed cut-offs: 25%, 50%, and 35%. The 25% cut-off is arbitrary; the 50% cut-off is used for describing lymphocyte-predominant breast cancer (LPBC) but is also considered arbitrary [17]. By using TILs as a continuous variable, the 35% cut-off was obtained by ROC curve analysis in the training set, with DFS at three years as the outcome variable.

The selection of the 3-year DFS as the outcome variable for cut-off assessment was based on the following: a) the follow up period of the last study (HE 10/08) is still short; b) the number of patients at risk at 5 years compared to 3 years were excessively reduced for both HE 10/05 and HE 10/08 studies, leading to overestimation of the risk and probably to biased estimates; c) the annual hazards analysis for the first 5 years showed that for HER2 positive in the pre-trastuzumab era and for TNBC patients the risk of recurrence after 3 years was greatly reduced (Table S4).

Associations among demographic, clinical and treatment characteristics, as well as among TILs cut-offs, were examined. Chi-square tests were used in order to examine possible associations among categorical variables. For testing categorical with continuous variables, the Mann-Whitney or the Kruskal-Wallis test was used, where appropriate. Time-to-event distributions were estimated using the product limit method. Kaplan-Meier curves and log-rank tests were used for comparing time to event distributions and evaluating DFS and OS differences, while univariate Cox analysis was used for and reporting hazard ratios. Univariate Cox with interactions was used in order to identify factors that differentiated TILs’ effect, while Firth correction for monotone likelihood was used for reporting hazard ratios in the case of subgroups with no events.

Survival data including median follow-up for each trial and for the entire population are given in Table S2. Given the large difference in the follow-up periods between the pre- (HE 10/97, HE 10/00) and the post-trastuzumab trials (HE 10/05, HE 10/08), two approaches were used regarding the predictive analysis: a) use of the original data, and b) use of the normalized data. The latter were obtained by truncating the follow-up time of the pre-trastuzumab trials down to the maximum follow-up time of post-trastuzumab trials; this modification was adapted as a further validation step for the results that were obtained with the original data.

Univariate analysis was conducted in the whole dataset, in the training set and in the validation set. Interactions between TILs with the above described BC subgroups and the nodal status were also examined. All univariate tests were two-sided, while significance level was set at α = 0.05. In multivariate analysis the clinicopathological parameters were chosen by backward elimination among the following: age (>50 vs. ≤50), tumor size (>2 cm vs. ≤2 cm), histological grade (I vs. II vs. III-Undifferentiated), positive nodes (≥4 vs. 0-3), treatment group (E-T-CMF vs. ET-CMF vs. E-CMF-Doc vs. E-CMF-T vs. E-CMF), trastuzumab administration (Yes vs. No), adjuvant hormone therapy (Yes vs. No) and subtypes (Luminal A/B vs. Luminal-HER2 vs. HER2-enriched vs. TNBC). Significance threshold for keeping a variable in the final model was set at α = 0.15. Multivariate analysis was conducted in the whole dataset, as well as in each BC subgroup.

The analysis was fully compliant with the reporting recommendations for tumor marker prognostic studies [23]. The SAS software was used for statistical analysis (SAS for Windows, version 9.3, SAS Institute Inc., Cary, NC, USA).

## SUPPLEMENTARY TABLES


